# Cost-effectiveness of Short-Course Radiation Therapy vs Long-Course Chemoradiation for Locally Advanced Rectal Cancer

**DOI:** 10.1001/jamanetworkopen.2019.2249

**Published:** 2019-04-12

**Authors:** Ann C. Raldow, Aileen B. Chen, Marcia Russell, Percy P. Lee, Theodore S. Hong, David P. Ryan, James C. Cusack, Jennifer Y. Wo

**Affiliations:** 1 Department of Radiation Oncology, University of California, Los Angeles; 2 Department of Radiation Oncology, MD Anderson Cancer Center, Houston, Texas; 3 Department of Surgery, University of California, Los Angeles; 4 Department of Radiation Oncology, Massachusetts General Hospital, Boston; 5 Department of Medical Oncology, Massachusetts General Hospital, Boston; 6 Department of Surgery, Massachusetts General Hospital, Boston

## Abstract

**Importance:**

Although both short-course radiotherapy and long-course chemoradiotherapy have been practiced in parallel for more than 15 years, no cost-effectiveness analysis comparing these 2 approaches in patients with locally advanced rectal cancer has been published.

**Objective:**

To analyze the cost-effectiveness of short-course radiotherapy vs long-course chemoradiotherapy for the treatment of patients with locally advanced rectal cancer.

**Design, Setting, and Participants:**

This economic evaluation used a cost-effectiveness model simulating 10-year outcomes for 1 million hypothetical patients aged 65 years with locally advanced rectal cancer treated with either short-course radiotherapy or long-course chemoradiotherapy, followed by surgery and chemotherapy. Utilities and probabilities from the literature and costs from the Healthcare Bluebook and Medicare fee schedules were used to determine incremental cost-effectiveness ratios. It was assumed that long-course chemoradiotherapy would result in higher rates of low anterior resection (LAR). To model preference-sensitive care, a 2-way sensitivity analysis was conducted in which the utilities of the no-evidence-of-disease (NED) states with LAR and abdominoperineal resection (APR) were simultaneously varied. The analysis was repeated for patients with distal rectal tumors. Analysis was conducted from January to October 2018.

**Exposures:**

Short-course radiotherapy and long-course chemoradiotherapy.

**Main Outcomes and Measures:**

Incremental cost-effectiveness ratios.

**Results:**

Short-course radiotherapy was the cost-effective strategy compared with long-course chemoradiotherapy (incremental cost-effectiveness ratio, $133 495 per quality-adjusted life-year). Two-way sensitivity analysis revealed that the cost-effective approach for a given patient depended on the utilities for the NED-LAR and NED-APR states. Assuming that a greater proportion of patients with locally advanced distal tumors undergoing long-course chemoradiotherapy (39%) would proceed to LAR compared with those treated with short-course radiotherapy (19%), long-course chemoradiotherapy was the cost-effective approach (incremental cost-effectiveness ratio, $61 123 per quality-adjusted life-year).

**Conclusions and Relevance:**

Short-course radiotherapy was the cost-effective strategy compared with long-course chemoradiotherapy for patients with locally advanced rectal cancer. The cost-effectiveness of short-course radiotherapy vs long-course chemoradiotherapy was sensitive to the utilities of the NED-LAR and NED-APR health states, highlighting the importance of care that is sensitive to patient preference. Long-course chemoradiotherapy was the cost-effective approach for patients with distal tumors.

## Key Points

**Question:**

What is the cost-effectiveness of short-course radiotherapy vs long-course chemoradiotherapy for patients with locally advanced rectal cancer?

**Findings:**

In this economic evaluation, short-course radiotherapy was the cost-effective strategy, with an incremental cost-effectiveness ratio of $133 495 per quality-adjusted life-year. However, for the subset of patients with distal tumors, long-course chemoradiotherapy was the cost-effective approach, with an incremental cost-effectiveness ratio of $61 123 per quality-adjusted life-year.

**Meaning:**

Patients with locally advanced rectal cancer should be treated with preoperative short-course radiation therapy unless they require tumor downstaging prior to resection, in which case long-course chemoradiotherapy is cost-effective.

## Teaser

This economic evaluation compares the cost-effectiveness of short-course radiotherapy vs long-course chemoradiotherapy for locally advanced rectal cancer.

## Introduction

Radiation therapy prior to total mesorectal excision has been shown to yield low rates of pelvic recurrence in the treatment of rectal cancer.^1,2,3,4,5^ Two radiation treatment paradigms have emerged as effective: (1) long-course chemoradiotherapy (LCRT) (50.4 Gy in 28 fractions with concurrent fluorouracil-based chemotherapy) followed by delayed total mesorectal excision and (2) short-course radiotherapy (SCRT) (25 Gy in 5 fractions) followed by immediate surgical resection. Recent prospective randomized trials have compared these 2 treatment approaches and suggested no difference in short-term disease outcomes.^3,4,5^

Although LCRT remains the standard of care in the United States, many countries treat locally advanced rectal adenocarcinoma with SCRT.^6^ Supporters of SCRT point to patient convenience, lower cost, and less acute radiation toxicity. Those favoring LCRT have emphasized increased likelihood of tumor downstaging, allowing for increased rates of sphincter preservation for low-lying tumors and R0 resection in the setting of threatened mesorectal fascia. Given the generally favorable outcomes for locally advanced rectal cancer, proponents of LCRT have noted the potential increased risk of late radiation-induced toxic effects in the setting of hypofractionated radiation. While the Trans-Tasman Radiation Oncology Group (TROG)^3^ and Bujko et al^5^ reported no significant differences in late toxic effects between these 2 regimens to date, the median follow-up of this study is limited.

In the existing health care system, high costs without proportional improvements in quality or outcome have prompted demands for change, and value-based models have been suggested. In health care, value, as defined by health outcomes achieved per dollar spent, provides a framework on which health systems may base their resource allocation decisions.^7^ The significant debate regarding the optimal neoadjuvant regimen for patients with locally advanced rectal cancer lends itself well to cost-effectiveness analysis. Cost-effectiveness analysis is a set of mathematical tools designed to compare the relative costs and benefits of treatment alternatives.^8^ Accordingly, the goal of this study was to perform a cost-effectiveness analysis to compare SCRT vs LCRT in patients with locally advanced rectal cancer.

## Methods

**Figure 1.**
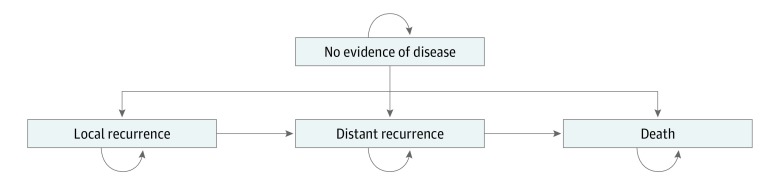
Markov Model of Treatment of Patients With Locally Advanced Rectal Cancer In our Markov model, hypothetical patients began in the well state with no evidence of disease, having undergone treatment for locally advanced rectal cancer. Patients then remained in this health state, proceeded to a local recurrence health state, or proceeded to a distant recurrence health state. At first transition to local recurrence, patients had the opportunity to undergo salvage treatment with the possibility of entering a salvaged local recurrence state. Patients could proceed to the death state owing to cancer or unrelated causes. The arrows indicate how the hypothetical patients could progress through the model.

We developed a Markov model to analyze the cost-effectiveness of SCRT vs LCRT for patients with locally advanced rectal cancer. In a Markov model, hypothetical cohorts of patients transition between different health states in fixed time increments and at defined probabilities (Figure 1). Markov models are used to model clinical situations in which a patient is at risk for a given event (eg, recurrence of rectal cancer) over an extended period. Patients start the model in a single health state. As the model cycles, the patients travel through time and may remain in their previous health state or transition to a different health state with a certain transition probability. This study was exempt from review by the University of California, Los Angeles, institutional review board and followed the Consolidated Health Economic Evaluation Reporting Standards (CHEERS) reporting guideline.^9^

### Strategies

Two treatment strategies were compared. In strategy 1, patients with locally advanced rectal cancer underwent LCRT followed by delayed total mesorectal excision (at 6-12 weeks) and adjuvant chemotherapy. In strategy 2, patients underwent SCRT followed by surgery 1 week later and subsequent adjuvant chemotherapy. For the base case analysis, we assumed 3-dimensional (3-D) conformal radiation treatment.

### Disease and Treatment Assumptions

Hypothetical patients began in 1 of several no-evidence-of-disease (NED) health states, having undergone either LCRT or SCRT with low anterior resection (LAR) or abdominoperineal resection (APR). Low anterior resection represents sphincter-sparing surgery with intrapelvic anastomosis, whereas APR is performed when the tumor is low-lying and sphincter sparing is not possible. Low anterior resection is typically accompanied by temporary ileostomy to allow adequate postoperative healing of the irradiated anastomosis and to minimize risk of anastomotic leaks. Ileostomy reversal is typically performed after completion of postoperative chemotherapy. Unlike LAR, APR requires a permanent colostomy. Hypothetical patients were distributed among these NED health states based on previously published rates.^1,2^ Patients then either remained in one of the NED health states or proceeded to the local recurrence or distant recurrence health states.^1,2^ We assumed that these rates were equivalent for SCRT and LCRT. At first transition to local recurrence, patients had the opportunity to undergo salvage treatment with possibility of entering a salvaged local recurrence state. Patients in the local recurrence and distant recurrence health states were able to transition to the death state as cancer-related deaths.^10^ Patients were also able to transition to the death state based on causes unrelated to cancer; the probability of dying of causes unrelated to cancer was based on Social Security actuarial life tables.^11^

We assumed that rates of late toxic effects were equivalent for SCRT and LCRT.^3^ This assumption was based on the TROG study,^3^ Bujko et al,^5^ and a recent meta-analysis,^12^ which reported no significant differences in late toxic effects between SCRT and LCRT. Given that these rates were assumed to be equivalent between the 2 strategies and would therefore not affect the incremental cost-effectiveness ratio (ICER) (the main outcome), the probabilities, cost, and utilities associated with these health states were excluded from the model. We further assumed that LCRT resulted in higher rates of LAR through tumor downstaging.^1^ Utilities were derived from a study explicitly evaluating patient utility values of stage II/III rectal cancer treated with resection, chemotherapy, and radiation therapy with or without permanent ostomy.^13^ Utilities and costs were discounted at 3% annually. Costs are presented in 2018 US dollars.

### Costs

A payer perspective was used to derive costs in this study. Radiation costs were calculated using 2018 National Medicare fee schedules^14^ and costs for hospital outpatient services on the basis of *Current Procedural Terminology* codes specific to radiation, including costs of consultation, simulation, weekly treatment management, treatment planning, and delivery. Medicare reimbursement was also used to calculate colostomy supply costs. Given the variability in postoperative care, hospital admission length, and anesthesia costs based on time in the operating room, which cannot be accurately calculated using National Medicare fee schedules, surgical costs were determined based on the fair price from the Healthcare Bluebook for rectal resection and closure of stoma.^15^ The fair price is the price that consumers should reasonably expect a medical service to cost if shopping for care. The fair price is calculated from actual amounts health plans have paid on claims and includes hospital services, physician services for the procedure and routine postoperative care, and anesthesia. Costs of acute toxic effects from treatment were not included as they were assumed to bias the model toward SCRT, with results to be taken as robust if SCRT was found to be cost-effective under this assumption (SCRT with early surgery is associated with lower severe acute toxicity than LCRT^3,4,5^). Table 1 depicts the probabilities, costs, and utilities used in the study.

**Table 1.** Probabilities, Utilities, and Costs Used in the Markov Model

Model Parameters	Value	Source and/or Note
Probabilities		
Local recurrence	0.71	Sauer et al,^2^ 2012 (10-y probability)
Distant recurrence	0.30	Sauer et al,^2^ 2012 (10-y probability)
Death from metastatic rectal cancer	0.86	American Cancer Society^10^ (5-y probability)
APR following long-course chemoradiation	0.17	Sauer et al,^2^ 2012
LAR following long-course chemoradiation	0.83	Sauer et al,^2^ 2012
APR following short-course radiation therapy	0.28	Sauer et al,^2^ 2012 (assumed based on postoperative group of trial)
LAR following short-course radiation therapy	0.72	Sauer et al,^2^ 2012 (assumed based on postoperative group of trial)
Utilities		
No evidence of disease after APR	0.50	Ness et al,^13^ 1999
No evidence of disease after LAR	0.59	Ness et al,^13^ 1999
Local recurrence	0.40	Expert opinion based on Ness et al,^13^ 1999
Distant recurrence	0.20	Ness et al,^13^ 1999
Death	0	Standard assumption
Costs, $		
APR	21 569	Healthcare Bluebook,^15^ 2018
LAR with ileostomy reversal	35 569	Healthcare Bluebook,^15^ 2018
Capecitabine (28-d supply at 1500 mg 2 times/d)	1890	Healthcare Bluebook,^15^ 2018
Colostomy supplies	3427	National Medicare fee schedules^14^
Long-course chemoradiation		
3-Dimensional conformal radiation therapy, including cost of capecitabine	19 311	National Medicare fee schedules^14^
Intensity-modulated radiation therapy, including cost of capecitabine	25 502	National Medicare fee schedules^14^
Short-course radiation therapy		
3-Dimensional conformal radiation therapy	7223	National Medicare fee schedules^14^
Intensity-modulated radiation therapy	7814	National Medicare fee schedules^14^

Abbreviations: APR, abdominoperineal resection; LAR, low anterior resection.

### Markov Model and Analysis

We developed a discrete-time Markov model to simulate 10-year outcomes for hypothetical patients aged 65 years (Figure 1). Analysis was conducted from January to October, 2018. The cycle length of the model was 1 year; the 10-year risks of local recurrence were derived from the German Rectal Cancer Study^1,2^ and were converted to 1-year probabilities to inform our model. The model was created and analyzed with TreeAge Pro 2017 (TreeAge Software, Inc). To ensure convergence in model outcomes, we simulated 1 million patients, and the history of events for each individual was tracked over the time span of the study. Our model was validated by comparing our model outputs with the expected local recurrence, distant recurrence, and overall survival rates of the German Rectal Cancer Study.^1,2^

We report the ICER when one strategy is more effective but more costly compared with the other. A treatment strategy with an ICER value less than the societal willingness to pay (WTP) is considered to be cost-effective.^8^ We defined cost-effectiveness at an ICER of $100 000 or less per quality-adjusted life-year (QALY).

Sensitivity analyses were used to evaluate the effect of adjusting the assumptions of the model. We performed 1-way sensitivity analyses varying key parameters. Clinical parameter ranges were based on data obtained via literature review where possible; otherwise, parameters were varied over a range of 0.5 to 1.5 times the base case value.^13^ To model preference-sensitive care, we conducted 2-way sensitivity analyses in which we simultaneously varied the utilities of the NED-APR and NED-LAR states. To explore whether our findings were sensitive to variations in surgical and radiation costs, we conducted 2 additional 2-way sensitivity analyses varying these costs. Because Medicare costs for radiotherapy may underestimate costs as compared with private payers, we performed an additional 2-way sensitivity analysis in which we varied the surgical costs assuming that radiation costs were 3 times higher than in the base case analysis, with SCRT costing $21 669 and LCRT costing $57 933. In addition, we performed a probabilistic sensitivity analysis with 100 000 samples to consider the combined uncertainty related to multiple parameters in the model. Beta distributions were used for probability and utility variables, and normal distributions were used for cost variables.

Our base case analysis assumed that all SCRT and LCRT were planned and delivered using 3-D conformal radiation treatment. To model the current practice of many radiation oncologists, we repeated the analysis assuming 3-D conformal treatment for LCRT but intensity-modulated radiation therapy for SCRT.

Our base case analysis included all patients eligible for the German Rectal Cancer Study,^1^ irrespective of tumor location within the rectum. To determine the cost-effectiveness of SCRT as compared with LCRT for patients with distal tumors only, we reran the analysis assuming that all patients had distal tumors. For this analysis, we assumed that 39% of patients who underwent LCRT where APR was initially deemed necessary were able to undergo LAR, compared with 19% of those undergoing SCRT.^1^

Finally, we also calculated the cost per permanent colostomy prevented and the number of patients needed to undergo LCRT to prevent a permanent colostomy. These values were calculated by the following formulas: cost per permanent colostomy prevented = (cost of strategy 1 − cost of strategy 2)/(number of colostomies in strategy 1 – number of colostomies in strategy 2); and number needed to treat with LCRT to prevent a permanent colostomy = 1/(proportion of patients with colostomy in strategy 1 – proportion of patients with colostomy in strategy 2).

## Results

We found that SCRT and LCRT were associated with QALYs of 4.72 and 4.79, respectively. In the base case analysis, SCRT was the cost-effective strategy compared with LCRT (ICER of LCRT vs SCRT, $133 495/QALY, greater than the WTP of $100 000/QALY) (Table 2).

**Table 2.** Incremental Cost-effectiveness Ratios for Each of the Treatment Strategies

Strategy	$		Effectiveness, QALY	Incremental Effectiveness	Incremental Cost-effectiveness Ratio, $/QALY
	Cost	Incremental Cost			
All patients					
SCRT (3-DCRT)	48 336	0	4.72	0	133 495
LCRT (3-DCRT)	58 369	10 033	4.79	0.08	
SCRT (IMRT)	48 926	0	4.72	0	125 632
LCRT (3-DCRT)	58 369	9443	4.79	0.08	
Distal tumors only					
SCRT (3-DCRT)	58 234	0	4.36	0	61 123
LCRT (3-DCRT)	66 587	8353	4.49	0.14	
SCRT (IMRT)	58 825	0	4.36	0	56 799
LCRT (3-DCRT)	66 587	7762	4.49	0.14	

Abbreviations: 3-DCRT, 3-dimensional conformal radiation therapy; IMRT, intensity-modulated radiation therapy; LCRT, long-course chemoradiotherapy; QALY, quality-adjusted life-year; SCRT, short-course radiation therapy.

**Figure 2.**
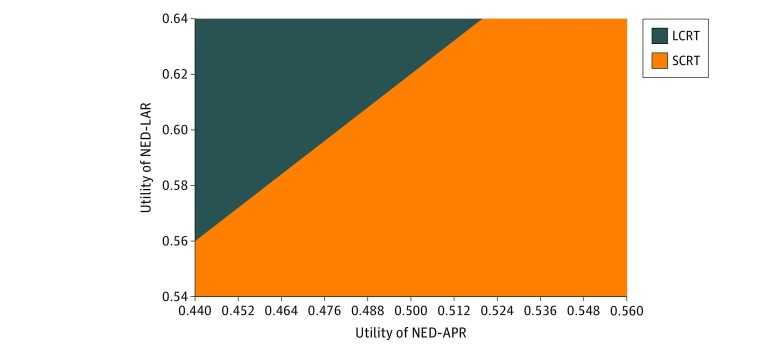
Two-Way Sensitivity Analysis (Willingness to Pay = $100 000) Varying the Utilities of the No-Evidence-of-Disease (NED) State After Low Anterior Resection (LAR) vs After Abdominoperineal Resection (APR) The utilities of the NED-LAR and NED-APR states were 0.59 and 0.50, respectively, in the basic model. To model preference-sensitive care, we conducted a 2-way sensitivity analysis in which we simultaneously varied the utilities of the NED-LAR and NED-APR states. The figure shows the most cost-effective approach (short-course radiotherapy [SCRT] in orange and long-course chemoradiotherapy [LCRT] in blue) depending on the utilities assigned to these 2 states. For example, if the utility of NED-APR is 0.45 and the utility of NED-LAR is 0.60, then LCRT is the cost-effective approach.

In the base case analysis, the utility of NED-LAR was 0.59 and the utility of NED-APR was 0.50. To model how different individuals value living in a health state with no evidence of disease with and without permanent ostomy, we performed 1-way sensitivity analyses varying the utilities of the NED-APR and NED-LAR states. On 1-way sensitivity analysis, LCRT became the cost-effective approach when the utility of NED-APR was below 0.47 (Table 3). On 1-way sensitivity analysis, LCRT became cost-effective when the utility of the NED-LAR was above 0.62 (Table 3). Two-way sensitivity analysis revealed that the cost-effective approach for a given patient depended on the utilities for the NED-LAR and NED-APR states. Figure 2 demonstrates the cost-effectiveness of SCRT vs LCRT given different utilities for the 2 health states.

**Table 3.** One-Way Sensitivity Analyses of Key Variables^a^

Parameters	Value	Tested Range	Lower Bound	Upper Bound	Threshold Value
Probabilities					
APR following LCRT	0.17	0.085-0.255	LCRT	SCRT	0.14
LAR following LCRT	0.83	0.415-1	SCRT	LCRT	0.86
APR following SCRT	0.28	0.14-0.42	SCRT	LCRT	0.31
LAR following SCRT	0.72	0.36-1	LCRT	SCRT	0.69
Local recurrence following SCRT	0.071 (10-y probability)	0.0355-0.1065 (10 y-probability)	SCRT	LCRT	0.104 (10-y probability)
Utilities					
No evidence of disease after APR	0.50	0.44-0.56^13^	LCRT	SCRT	0.47
No evidence of disease after LAR	0.59	0.54-0.64^13^	SCRT	LCRT	0.62
Costs, $					
APR	21 569	10 785-32 354	SCRT	SCRT	NA
Colostomy	3427 (annual)	1714-5141	SCRT	SCRT	NA
LAR with ileostomy reversal	35 569	17 785-53 354	SCRT	SCRT	NA
LCRT, including cost of capecitabine	19 311	9655-28 997	LCRT	SCRT	16 793
SCRT	7223	3612-10 835	SCRT	LCRT	9741

Abbreviations: APR, abdominoperineal resection; 3-DCRT, 3-dimensional conformal radiation therapy; LAR, low anterior resection; LCRT, long-course chemoradiotherapy; NA, not applicable; SCRT, short-course radiation therapy.

^a^ One-way sensitivity analyses examine the effect of altering 1 value in the model on the model’s results. The text under “Lower Bound” (SCRT vs LCRT) denotes the strategy that was more efficacious when the parameter was varied to the lowest value in the specified range. The text under “Upper Bound” (SCRT vs LCRT) denotes the strategy that was more efficacious when the parameter was varied to the highest value in the specified range. The threshold value denotes the value at which the cost-effective strategy changes within the tested range.

To model the current practice of many radiation oncologists, we performed an analysis where 3-D conformal treatment was used for LCRT but intensity-modulated radiation therapy was used for SCRT. Under this assumption, SCRT remained the cost-effective strategy (ICER of $125 632/QALY) (Table 2).

To further explore whether our findings were sensitive to variations in cost between SCRT and LCRT, we conducted 1-way sensitivity analyses and a 2-way sensitivity analysis varying these costs. Table 3 reveals that SCRT remained the cost-effective approach unless the cost of LCRT fell below $16 793. Two-way sensitivity analysis showed that the cost-effective approach varied depending on the relative costs of SCRT and LCRT (eFigure 1 in the Supplement). However, SCRT remained the cost-effective approach for the majority of the cost ranges tested.

To explore whether our findings were sensitive to variations in cost between APR and LAR, we conducted 1-way sensitivity analyses and a 2-way sensitivity analysis varying these costs. Table 3 reveals that over the plausible range of surgical costs, SCRT remained the cost-effective approach. Two-way sensitivity analysis confirmed that SCRT remained the cost-effective approach for different costs of APR and LAR over the range tested. Even when we assumed that the radiation costs were 3 times higher than in the base case analysis, SCRT remained the cost-effective approach for the different costs of APR and LAR over the range tested.

In the base case analysis, we assumed that local recurrence rates were equivalent for SCRT and LCRT, with a 10-year probability of 7.1%.^1,2,3,4,5^ To explore whether our findings were sensitive to variation in the benefit of SCRT vs LCRT radiation, we conducted a sensitivity analysis varying the probability of local recurrence conferred by SCRT. We found that LCRT became the cost-effective approach when the 10-year probability of local recurrence risk after SCRT was 10.4% or higher (Table 3).

To assess the effect of varying all uncertain parameters of the model simultaneously, we conducted a probabilistic sensitivity analysis using Monte Carlo simulation of 1 000 000 trials. As eFigure 2 in the Supplement indicates, SCRT was cost-effective in 63.5% of trials at a WTP of $100 000/QALY.

Because LCRT was not cost-effective, we also examined the costs per permanent colostomy prevented as compared with SCRT. The cost per permanent colostomy prevented was $91 155. The number needed to treat with LCRT per permanent colostomy prevented was 8.23.

We repeated the analysis with all patients having distal tumors. In this analysis, SCRT and LCRT were associated with QALYs of 4.36 and 4.49, respectively, and LCRT was the cost-effective strategy (ICER of LCRT vs SCRT, $61 123/QALY, less than the WTP of $100 000/QALY) (Table 2). On sensitivity analysis, we found that LCRT was the cost-effective approach when at least 13.9% more patients undergoing LCRT vs SCRT had tumor downstaging sufficient for LAR.

## Discussion

The treatment of locally advanced rectal adenocarcinoma is unique in that 2 neoadjuvant approaches, SCRT and LCRT, have been studied in parallel and found effective in improving local control.^1,2,16^ To date, there have been 2 prospective randomized trials that have compared these 2 radiation treatment approaches.^3,4,5^

Although the convenience and cost benefit of receiving 5 fractions of radiation therapy instead of 28 are self-evident, LCRT is thought to result in higher rates of sphincter preservation. Under the stated disease and treatment assumptions, we found that SCRT was the cost-effective strategy compared with LCRT for patients with locally advanced rectal adenocarcinoma. Sensitivity analyses that varied costs of surgery and costs of radiation therapy over a reasonable range showed that SCRT was the higher-value treatment over the vast majority of the ranges tested.

There were several scenarios in which LCRT became the cost-effective approach compared with SCRT. For instance, we found that LCRT became the cost-effective approach when it was associated with a 3.3% absolute improvement in locoregional disease control. However, it is critical to note that currently available published data do not support any degree of improved locoregional control of LCRT over SCRT.

We also found that lowering the utility of the NED-APR health state by more than 0.03 below the utility assumed in the base case drove the ICER to favor LCRT. In other words, the benefit of LCRT depends on how patients value living with vs without a permanent ostomy. We used published population-based utility values to inform the analyses of our model.^13^ However, utilities may vary significantly among patients, and such variations can create different optimal treatments for individual patients.^8^ These results confirm the importance of eliciting patient preferences in decision making, as preference determination would allow for more tailored and cost-effective treatment.

Although SCRT was the higher value treatment when including all patients with locally advanced rectal cancer, we reran the analysis with the assumption that all patients had distal tumors that would require APR prior to undergoing radiation. For this analysis, we assumed that 39% of patients who underwent LCRT where APR was initially deemed necessary were able to undergo sphincter-preserving surgery as compared with 19% of those undergoing SCRT.^1,2^ Our results therefore suggest that LCRT is the cost-effective approach for patients with distal tumors.

Although LCRT was not cost-effective in the base case scenario, it reduced the number of patients needing a permanent ostomy. Quality of life is often impaired in patients with a stoma. In a study that evaluated LAR vs APR in patients with rectal cancer using patient-reported outcomes in the National Surgical Adjuvant Breast and Bowel Project randomized trial R-04,^17^ patients who underwent APR reported worse body image and worse sexual enjoyment at 1 year as compared with patients who underwent LAR. In a different study that included 336 patients with rectal cancer with a stoma and 117 patients without a stoma, patients with a stoma had significantly worse mental health, body image, and sexual function compared with those without a stoma.^18^ In addition, those patients with a stoma experienced more fatigue and loss of appetite than those who underwent LAR. Thus, whether potentially avoiding a stoma by undergoing LCRT is a benefit sufficient to justify its increased cost should be openly discussed by all stakeholders.

### Limitations

Several limitations of our study must be acknowledged. First, we made some simplifying assumptions about the natural history and treatment of disease to specify a finite number of clinical events. Second, we may not account for differences in late toxic effects of SCRT vs LCRT with modern techniques, as follow-up data are limited.^3^ Although late toxic effect rates in the TROG study,^3^ Bujko et al,^5^ and a recent meta-analysis^12^ were no different, basic radiobiologic principles dictate that larger fraction size has a potential for higher risk of late toxic effects that can manifest in subsequent decades. However, despite limited follow-up at this time, although nonsignificant, the absolute rate of late toxic effects in the TROG study appears lower for SCRT than for LCRT, giving a sense that it is unlikely that the longer-term follow-up for the TROG data will show significantly higher late toxic effects in the SCRT group. Given that the time frame for this model was 10 years, we felt that our assumption was reasonable. Additionally, it is important to acknowledge that there are many factors that should play a role in determining the optimal treatment strategy for any individual patient with rectal cancer. Some of these factors, including subjective impact of radiation therapy on ease of achieving a complete total mesorectal excision resection and impact of radiation on surgically induced nerve injury, are beyond the scope of our analysis but should be discussed in the context of optimal care. Consequently, the treatment paradigm of rectal cancer is best decided in a multidisciplinary tumor clinic setting, with robust discussion between the surgeon, medical oncologist, and radiation oncologist. There are newer emergent treatment strategies for locally advanced rectal cancer, including definitive chemoradiation (with nonoperative management) and total neoadjuvant therapy, both in the setting of SCRT and LCRT, that are out of the scope of this analysis.^19,20,21^

## Conclusions

We found that among patients with locally advanced rectal cancer, SCRT was the cost-effective approach compared with LCRT given current parameters. We found that LCRT became the cost-effective approach when the utility of a patient with permanent colostomy was lower by more than 0.12 than that of a patient without a permanent colostomy. Therefore, physicians should elicit individual patient preferences to maximize quality-of-life outcomes. For the subset of patients with distal tumors, LCRT was the cost-effective approach.

## Notes

Supplement. **eFigure 1.** Two-Way Sensitivity Analysis (Willingness to Pay = $100,000) Varying the Costs of SCRT and LCRT **eFigure 2.** Probabilistic Sensitivity Analysis (1,000,000 Trials) Varying All Uncertain Parameters of the Model Simultaneously
