# Supplementary material for: Cost-effectiveness of Short-Course Radiation Therapy vs Long-Course Chemoradiation for Locally Advanced Rectal Cancer
Source: JAMA Netw Open. 2019 Apr 12;2(4):e192249. doi: 10.1001/jamanetworkopen.2019.2249 (PMC6481445; doi:10.1001/jamanetworkopen.2019.2249)
Supplement: Supplement. — eFigure 1. Two-Way Sensitivity Analysis (Willingness to Pay = $100,000) Varying the Costs of SCRT and LCRT eFigure 2. Probabilistic Sensitivity Analysis (1,000,000 Trials) Varying All Uncertain Parameters of the Model Simultaneously [file jamanetwopen-2-e192249-s001.pdf]

Supplementary Online Content

Raldow AC, Chen AB, Russell M, et al. Cost-effectiveness of short-course radiation therapy vs long-course chemoradiation for locally advanced rectal cancer. *JAMA Netw Open*. 2019;2(4):e192249. doi:10.1001/jamanetworkopen.2019.2249

eFigure 1. Two-Way Sensitivity Analysis (Willingness to Pay = $100,000) Varying the Costs of SCRT and LCRT
eFigure 2. Probabilistic Sensitivity Analysis (1,000,000 Trials) Varying All Uncertain Parameters of the Model Simultaneously

This supplementary material has been provided by the authors to give readers additional information about their work.

© 2019 Raldow AC et al. *JAMA Network Open.*

eFigure 1. Two-way sensitivity analysis (willingness to pay = $100,000) varying the costs of SCRT and LCRT. The costs of SCRT and LCRT were $7,223 and $19,311, respectively, in the basic model. The figure shows the most cost-effective approach (SCRT in red or LCRT in blue) depending on the costs of SCRT and LCRT. Abbreviations: LCRT, long-course chemoradiation; SCRT, short course radiation therapy

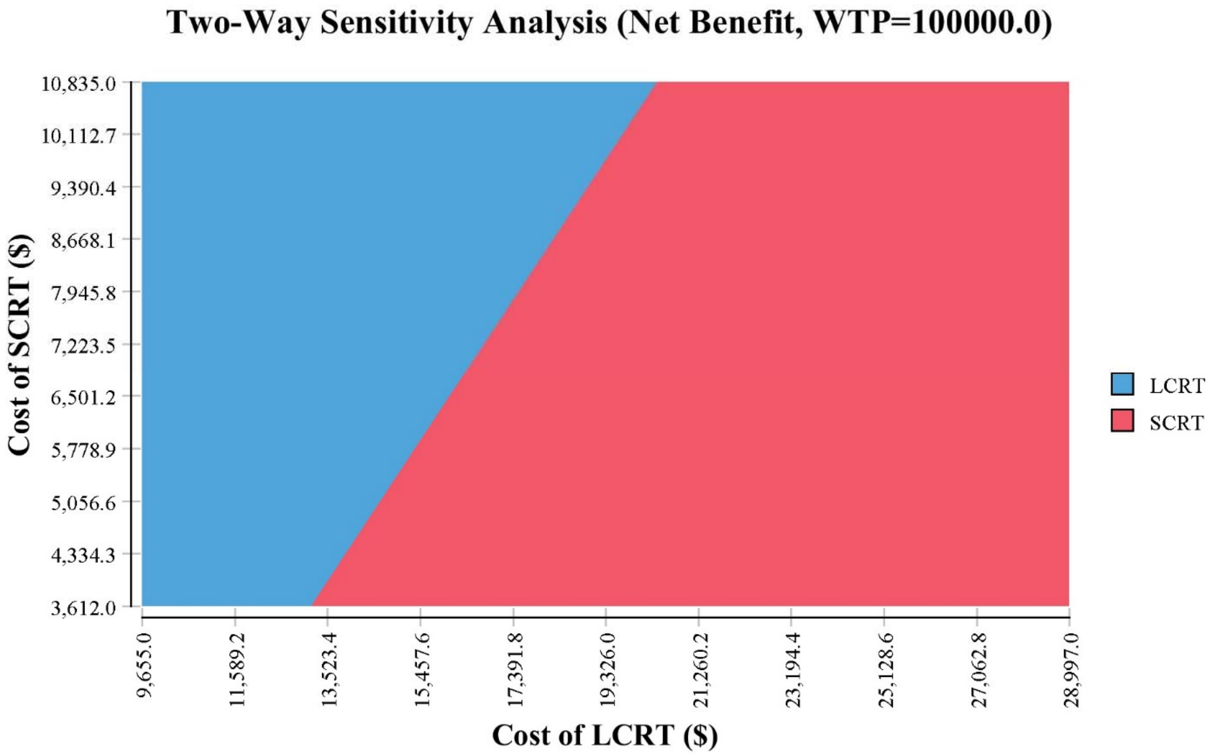

© 2019 Raldow AC et al. *JAMA Network Open.*

eFigure 2. Probabilistic sensitivity analysis (1,000,000 trials) varying all uncertain parameters of the model simultaneously. The graph indicates the percentage of trials in which a strategy is cost-effective at various willingness to pay thresholds. For example, at a willingness to pay of $100,000 per quality-adjusted life year, SCRT was cost effective in 63.5% of simulations. Abbreviations: LCRT, long-course chemoradiation; SCRT, short course radiation therapy

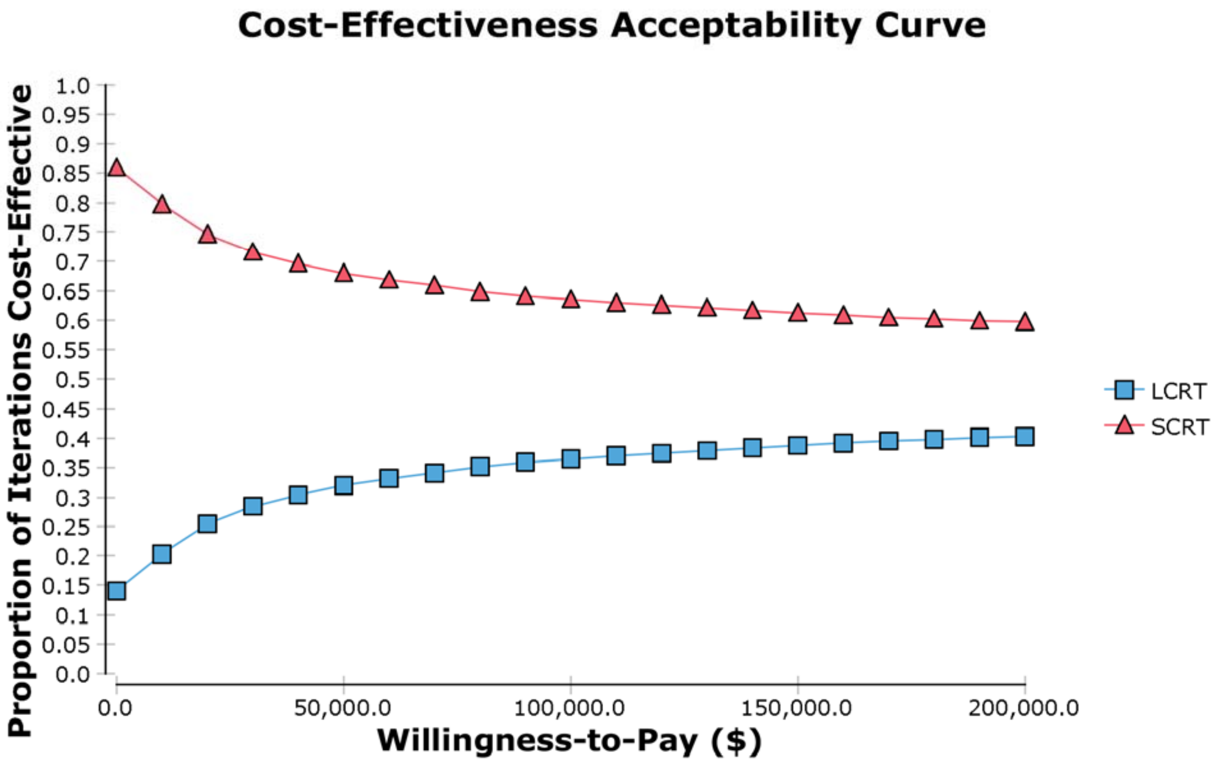

© 2019 Raldow AC et al. *JAMA Network Open.*
